# Psychological adverse effects of COVID-19 pandemic on health service providers: an online survey

**DOI:** 10.1186/s43045-022-00253-6

**Published:** 2022-10-26

**Authors:** Mohammad Hossein Somi, Ali Fakhari, Hosein Azizi, Habibeh Barzegar, Sanaz Norouzi, Vahab Aslrahimi, Mostafa Farahbakhsh

**Affiliations:** 1 Liver and Gastrointestinal Diseases Research Center, Tabriz University of Medical Sciences, Tabriz, Iran; 2 Research Center of Psychiatry and Behavioral Sciences, Tabriz University of Medical Sciences, Tabriz, Iran; 3 Unit for Mental Health, Tabriz University of Medical Sciences, Tabriz, Iran; 4 Road Traffic Injury Research Center, Tabriz University of Medical Sciences, Tabriz, Iran

**Keywords:** Health service provider, COVID-19, Depression, Anxiety, Iran

## Abstract

**Background:**

In the current situation of the COVID-19 pandemic, health service providers (HCPs) suffered from mental health consequences such as depression, anxiety, fear, and post-traumatic stress disorder (PTSD). The study aimed to evaluate the adverse psychological impacts of the COVID-19 pandemic on HSPs at the Tabriz University of Medical Science, North West of Iran. An online survey was conducted to assess the psychological adverse effects of COVID-19 during the pandemic of COVID-19 from May 2021 to February 2022. Psychological adverse effects including depression, anxiety, PTSD, and fear were measured using valid instruments. Overall, 298 HSPs responded to the questionnaires. Descriptive and multiple logistic regression analyses with crude and adjusted ORs were used to estimate mental health adverse effects.

**Results:**

The overall prevalence of depressive symptoms, major depression disorder (MDD), anxiety, and probable PTSD were 58%, 9.1%, 61.7%, and 15%, respectively. The fear of COVID-19 scale (mean) was 17.6± 6.2. We found mental health adverse effects were higher in HSPs who worked in the treatment and health sections than HSPs in the support section. Having a history of mental disorder, HSP type (health and treatment), and female sex had a statistically significant association with MDD and depressive symptoms.

**Conclusions:**

Mental health problems were high in HSPs. The study highlights the demand for support systems and appropriate interventions for improving HSPs’ mental health and well-being during the COVID-19 pandemic.

## Background

Severe acute respiratory syndrome coronavirus 2 (SARS-CoV-2), commonly named coronavirus disease 2019 (COVID-19) [1, 2], has strongly affected the performance of healthcare systems around the world [3]. It is clear that during this challenging time, people may be feeling fear, depression, anxiety, and worry due to COVID-19 morbidity and mortality and the continuous shifting alerts of the virus [4].

During the COPVID-19 pandemic, health service providers (HCPs) suffered from huge mental health distress in healthcare settings [5]. Given that HSPs are providing direct care for COVID-19 patients, they are more possible to be infected than other groups of people [6]. This makes them fear the virus transmission, worry for family health, personal isolation, trust in and support from their institute, and stigma [7–9].

Consequently, HSPs will be under overwhelming mental health concerns which may suspect various psychological consequences, such as fear, depression, anxiety, and stress. Evidence indicated that pooled prevalence for anxiety varied from 45% to 69% and depression from 38% to 60%, and acute stress disorder was 31% to 82% [10].

In a study conducted in Ethiopia during the COVID-19 pandemic, the prevalence of depression, anxiety, and stress were 58.2%, 64.7%, and 63.7%, respectively [8]. Likewise, in China, 53.8% of the participants suffered from psychological problems, 16.5% depressive symptoms, 28.8% anxiety symptoms, and 8.1% reported stress [11]. Similarly, in the study conducted among nurses in Taiwan, 11% of them suffered anxiety, depression, and somatization during the pandemic [12].

Several studies have been performed on the mental health of HSPs during the COVID-19 pandemic in various countries [13–16]. However, there is poor evidence in East Azerbaijan Province, especially regarding depression, anxiety, post-traumatic stress disorder (PTSD), and fear of the virus. The results of this study can provide imperative information to support healthcare managers and the provision of mental health services for HSPs. Furthermore, a better understanding of the mental health impacts of COVID-19 on HSPs is significant to identify appropriate interventions.

## Methods

### Study design and sampling

An online cross-sectional survey was performed to assess the psychological consequence and worries of COVID-19 pandemic in health service providers (HSPs) at Tabriz University of Medical Sciences during the pandemic from May 2021 to February 2022. The target population was all employed HSPs at Tabriz University of Medical Sciences. The study samples were included through stratified random sampling and proportional to the size of the University’s three Vice Chancellors and the size of health service centers. At first, we categorized all HSPs into three strata including “health and education,” “treatment,” and “supportive,” and then samples were assigned based on the size (number of HSPs) of each stratum. Within the stratum, samples were considered for each unit in proportion to the size of that stratum. Health and Education providers who worked under health Vice Chancellors provided first-line healthcare services through primary health care (PHC) in Iran. Apart from screening centers, they are less in direct contact with COVID-19 patients. They are often known as community health workers in Iran [6]. HSPs who worked under treatment Vice Chancellors include physicians and non-physicians in hospitals, and most of those are in direct contact with COVID-19 patients. HSPs who worked under support Vice Chancellors included non-medical providers who present support services for other cadres including manpower supply, resource management, finance, recruitment, and cleaning and physical services.

The sample size was determined by considering a confidence level of 95%, *α*=0.05%, *P*=0.4, *d*=0.1p, and 10% compensation due to non-respondents; the total sample size was 300 subjects.

### Eligibility

Inclusion criteria considered employed HSPs at the Tabriz University of Medical Sciences with at least 1 year experience and included any type of employment such as formal, contractual, or temporary. Exclusion criteria have also considered non-informed consents.

### Data collection

To prevent the spread of COVID-19 infection and easy access, an online (platform) survey was conducted for assessing the psychological outcomes and implications of the COVID-19 pandemic on HSPs. The online link of the questionnaire was sent to the participants through the university website that was accessible to all HSPs, electronic networks (WhatsApp), official letters, and/or emails.

### Measures

We assessed COVID-19-related health concerns and mental health consequences including depression, anxiety, post-traumatic stress disorder (PTSD), and phobia. In addition to the assessment of the psychological adverse effects of COVID-19 on HSPs, we also asked about their history of depressive disorders and antipsychotic use and referred them to a psychiatrist before and/or during the COVID-19 pandemic through self-reporting. In this way, we are able to compare a load of referrals to receive psychiatric services in the pre- and post-COVID-19 pandemic period among HSPs.

*Patient Health Questionnaire (PHQ-9)* Depression Scale [17] was used to assess both major depression and sub-threshold depressive disorder in the HSPs [18]. The PHQ-9 measures depressive symptoms and severity over the past 2 weeks, and the scoring system is based on a 4-point scale: 0 (*not at all*), 1 (*several days*), 2 (*more than half the days*), and 3 (*nearly every day*) based on previous study [19]. The total scores of the tool ranged between 0 and 27. MDD is suggested if of the 9 items, “5 or more are checked as at least ‘more than half the days’” and/or “either item 1 or 2 is checked as at least ‘more than half the days.’” Threshold depressive symptom is suggested if “Of the 9 items, between 2 to 4 are checked as at least ‘more than half the days’” and/or “either item 1 or 2 is checked as at least ‘more than half the days.’” The psychometric properties of the Persian version of PHQ9 were confirmed in various groups of populations in Iran [20–22].

General Anxiety Disorder-7 item (GAD-7) scale used to evaluate the general anxiety symptoms in HSPs. The tool involves 7 items that assess GAD symptoms and their severity. Respondents rated their level of agreement with the statements using a 4-point scale: 0: not at all, 1: several days, 2: more than half of the days, and 3: nearly every day. The score range is between 0 to 21; the higher GAD-7 score indicates greater symptom severity [23]. The reliability and validity of the Persian version of GAD-7 were confirmed in previous studies in Iran among several target groups [24, 25]. The Cronbach’s alpha for the Persian version of the GAD-7 scale was 0.88 [26]. GAD-7 is the frequently used screening measure for generalized anxiety symptoms in different patient groups [27].

*Fear of COVID-19 scale (FCV-19S), Persian version* [28, 29], is a 7-item scale that measures the fear of COVID-19. Each item on the scale is responded to using a 5-point, Likert-type scale ranging from 1 (strongly disagree) to 5 (strongly agree). A total score could be calculated by summing up each item score (ranging from 7 to 35).

The primary care post-traumatic stress disorder for DSM-5 (PC-PTSD-5) scale [30] was used to evaluate respondents with low and probable PTSD over the past month. This tool involved a 5-item and begins with an item designed to measure whether the HSP has had any experience with traumatic events. If a HSP rejects all exposure, the PTSD-5 is complete with a score of 0. However, if a HSP indicates that she/he has experienced a traumatic event over the course of their life, the respondent is instructed to respond to five additional *yes/no questions* about how that trauma exposure has affected them. Probable PTSD is suggested if: of the 5 items, 4 or more items were positive (yes), and low probable is suggested if of the 5 items, 3 items were positive (yes) [31].

### Statistical analysis

The SPSS software (version 19.0, Chicago, IL, USA) was carried out for data analysis, Kolmogorov-Smirnov test to check data normality, chi-square (*χ*^2^) test to assess the relationship between dichotomous and nonparametric variables, independent *t*-test and Mann-Whitney for comparing parametric variables between two groups of with psychological disorders and without. Multiple logistic regressions [32] were used to estimate the adjusted odds ratio (OR) with a 95% confidence interval (CI) for the risk factors associated with psychological disorders. In all tests, the confidence interval was considered 95% and *P*-value <0.05 was significant.

## Results

Table 1 shows the baseline characteristics of the HSPs. Altogether, 298 HSPs responded to the survey. Of those, 192 (64.4%) were female. The mean age and work experiences of the respondents were 40.51 and 15.0 years, respectively. Regarding cadre type, respondents were 41.3% in the support section, 38.3% in the health section, and 20.5% in hospitals (section of treatment).

**Table 1:** The baseline characteristics of the study participants (health service providers)

Variables*	Health service providers (***n***= 298)	%
**Age**		
Mean ± SD	40.51 ± 8.70	
**Work history (year)**		
Mean ± SD	15.0 ± 8.70	
**Sex**		
Female	192	64.4
Male	106	35.6
**Marital status**		
Single	45	15.2
Married	253	48.8
**Health service provider type** (Cadre type)		
Health service cadre	114	38.3
Hospital service cadre	61	20.5
Support and resource development	123	41.3
**Educational level**		
Non-academic (under diploma)	31	10.4
High diploma	28	9.4
Bachelor	131	44.0
Master	71	23.8
Doctor and/or higher	37	12.4

*Self-reported

Table 2 demonstrated the prevalence and distribution of mental health disorders among health service providers. Overall, 34 (11.4%) of the providers self-reported a history of any mental health disorders. Before the COVID-19 pandemic, 10.4% of providers were presented to psychiatrists while during the COVID-19 pandemic, this measure was 14.1%.

**Table 2:** Distribution of mental disorder before and during the COVID-19 pandemic among HSPs at Tabriz University of Medical Sciences

Variables	Health service providers (***n***= 298)	%
**History of any mental disorder (prevalence of life)**		
Yes	34	11.4
No	264	88.6
**Referred to a psychiatrist** (before COVID-19)		
Yes	31	10.4
No	267	89.6
**Referred to a psychiatrist** (during COVID-19)		
Yes	42	14.1
No	256	85.9
**Having any mental disorder currently**		
Yes	64	21.5
No	234	78.5
**Mental disorder type** (at currently)		
Depression	24	37.5
Anxiety	15	23.43
Obsessive-compulsive disorder (OCD)	2	0.03
Other	23	36.0
**Received any antipsychotics** (due to the COVID-19 pandemic)		
Yes	24	8.0
No	274	92.0

More than 21% of the respondents self-reported any current mental health disorders. Of those, 37.5% and 23.4% of the providers reported depressive and anxiety disorders, respectively. Moreover, during the COVID-19 pandemic, 24 (8.0%) of the providers had received antipsychotic drugs due to COVID-19 worries and risks.

Table 3 shows the common mental health symptoms in health service providers at Tabriz University of Medical Sciences in the context of the COVID-19 pandemic. We found that 27 (9.1%) and 73 (24.5%) of respondents had MDD and threshold depressive syndrome, respectively. Altogether, 58% of respondents had any levels of depressive symptoms. The mean score of anxiety was 8.34 in HSPs who worked in treatment (hospitals), 7.18 in HSPs who worked in health, and 5.78 in HSPs who worked in the support section. Overall, 61.7% of respondents had anxiety symptoms. There were significant differences regarding anxiety scores between HSPs groups (*P*=0.022). More than 50% of the HSPs had mild anxiety symptoms while 3.7% of them had severe anxiety symptoms.

**Table 3:** COVID-19-related common mental health symptoms in health service providers at Tabriz Uni Med Sci

Variables			Health service providers (***n***= 298)					***P***-value
			Health	Treatment	Support	Total		
**Major depressive disorder** (MDD)^**a**^	Yes		14	7	6	27 (9.1)		0.052
	No		100	54	117	271 (90.9)		
**Threshold depressive syndrome**^**b**^	Yes		31	14	28	73 (24.5)		0.432
	No		83	47	95	225 (75.5)		
**Depressive symptoms**	Non-minimal (0–4)		44	22	59	125 (42.0)		0.477
	Mild (5–9)		26	19	34	79 (26.5)	58.1%	
	Moderate (10–14)		25	13	17	55 (18.5)		
	Moderately severe (15–19)		10	3	67	20 (6.7)		
	Severe (20–27)		9	4	6	19 (6.4)		
**Probable PTSD** (positive for 4 or more items of the 5 items)	Yes		20	10	14	44 (14.8)		0.370
	No		94	51	109	254 (85.2)		
**Low probable PTSD** (positive for 3 items of the 5 items)	Yes		30	13	26	69 (23.2)		0.603
	No		84	48	97	229 (76.8)		
**Phobia**	Mean ± SD					17.65 ± 6.27		0.248
	Scale	0–7	10	1	4	15 (5.0)		
		8–15	30	22	46	98 (32.9)	95%	
		16–23	55	26	57	138 (46.3)		
		24–31	16	10	12	38 (12.8)		
		≥ 32	3	1	4	8 (2.7)		
**Anxiety**	Mean ± SD		7.18± 5.7	8.34±5.9	5.78±4.9	6.96 ± 5.60		0.022
	Scale	Minimal: 0–4	72	16	42	130 (52.7)		
		Mild: 5–9	64	14	34	112 (27.9)	210 (61.7)	
		Moderate: 10–14	35	9	13	57 (15.1)		
		Severe: 15–21	24	10	7	41 (3.7)		

^a^MDD is suggested if: of the 9 items, 5 or more are checked as at least “more than half the days” and/or either item 1 or 2 is checked as at least “more than half the days”

^b^Threshold depressive symptoms are suggested if: of the 9 items, between 2 and 4 are checked as at least “more than half the days” and/or either item 1 or 2 is checked as at least “more than half the days”

Regarding PTSD, 44 (14.8%) and 69 (23.2%) of respondents reported probable and low probable PTSD, respectively. Concerning phobia symptoms, the average score of fear of COVID-19 (7-item scale) was 17.65 ± 6.27.

To estimate risk factors for MDD in the presence of HSP types, bivariate, and multivariate binary logistic regression analyses were carried out (Table 4). We found the risk of MDD among healthcare providers who worked in the health and treatment (hospitals) sections is 2.36 and 2.67 times higher than among healthcare providers who worked in the support section. In the final analysis, history of mental illness, types of HSPs, sex, and educational level were found to be associated with MDD.

**Table 4:** Association between MDD and health service provider type during the COVID-19 pandemic by multiple logistic regression analysis after adjusting for the potential confounders

Variables	Crude OR; 95% CIs	***P***-value	Adjusted OR; 95% CIs	***P***-value
**Healthcare type**				
Support	1	1	1	1
Health	2.73 (1.01–7.37)	0.047	2.36 (0.85–6.57)	0.100
Treatment	2.53 (0.81–7.88)	0.110	2.67 (0.84–8.50)	0.097
**History of mental disorder**				
No	1	1	1	1
Yes	3.16 (1.22–8.16)	0.027	3.14 (1.19–8.30)	0.021
**Sex**				
Male	1	1	1	1
Female	2.01 (1.02–4.50)	0.042	2.25 (0.82–6.32)	0.122
**Age**				
<30	1.48 (0.74–2.97)	0.265	1.54 (0.74–3.24)	0.250
>30	1	1	1	1
**Marital status**				
Single	1	1	1	1
Married	1.0 (0.49–2.10)	0.982	1.06 (0.48–2.33)	0.887
**Educational level**	0.73 (0.57–0.93)	0.012	0.72 (0.54–0.95)	0.024

## Discussion

To the best of our knowledge, the current study is the first related to the HSP mental health adverse effects of the COVID-19 pandemic in East Azerbaijan Province, Iran. We found that the COVID-19 pandemic affected the mental health of HSPs.

In this study, 58% of the HSPs had depressive symptoms related to the COVID-19 pandemic. In support of our findings, Asnakew et al. [8] in Ethiopia and Libya [33] were found; 58% of healthcare providers also had depressive symptoms. According to the current study, 9% of HSPs had MDD, and 24.5% had threshold depressive syndrome related to the COVID-19 pandemic. The prevalence of depressive symptoms on HSPs in the current study was higher than the studies performed in China 44% [34], 15.4% [12] Spanish 46% [35] systematic review studies 22.8% [36], and India 11.4% [37]. However, in the present study, the prevalence of depression was lower than the study conducted in Turkey 77.6% [38]. The cause for this difference might be the time of the study conducted in the context of the pandemic, the epidemic curve, and also the study’s sample size.

In this survey, the prevalence of MDD was 9.1%. The prevalence of MDD was found 8% in young adults of East Azerbaijan Province in 2020 [19]. Healthcare providers who worked in treatment and health sections showed a positive association with MDD and depression. Moreover, those who had a history of mental health problems, female sex, and had low educational levels were associated with MDD. In a study conducted in China, the prevalence of depressive symptoms (moderate and severe) was 18% [39]. The prevalence of MDD in the general population was reported at 3.6% according to a population-based study in South Korea [40], Danish 3.3%, and review studies [41] 4.7% around the world. In Iran, the prevalence of MDD was estimated at 4.1% (3.1–5.1), in a systematic review and meta-analysis [42]. An increase in the prevalence of depressive symptoms can elevate the probability of suicidal behaviors and suicide re-attempt [43–46].

In our study, the overall prevalence of anxiety symptoms were in line with the studies carried out in Ethiopia 64.7% [8] and Turkey 60.2% [47]. However, it was higher than the studies performed in Spain 58.6% [48] and China 44.6% [49] and 38% [50].

Concerning other mental health disorders, 15% of HSPs had probable PTSD, and 23.2% had low probable PTSD. Furthermore, the average score of fear scale related to COVID-19 was 16.65. Similarly, a study in Egypt found the mean of the fear of COVID-19 scale was 17.7 during the COVID-19 pandemic.

The findings in this study were in line with the studies conducted in Egypt. Abdelghani M et al. [51] found there was a robust correlation between HSPs who perceived fears and higher burnout symptoms. Likewise, a population-based study in Canada [52] indicated that HSP stigmatization is associated with COVID-19 stress syndrome. Similarly, a study in Bangladesh [53] found nearly over 1/4 of the HSPs had depression and was significantly related to COVID-19 fear.

In a systematic review and meta-analysis [54] that was pooled across 65 studies involving 97,333 healthcare workers across 21 countries, the prevalence of PTSD was 21.5% (95% CI, 10.5–34.9%).

Correspondingly, the prevalence of probable PTSD was in line with a review study (16.7%) [16]. This study found negative emotions and threats and/or physical tension are reliable predictors of PTSS. HSPs who suffered from higher levels of PTSS scored positively for insomnia and exhibited significantly higher PTSD.

## Conclusions

The study findings demonstrated that HSPs are affected by the high level of depression, PTSD, fear, and anxiety during the COVID-19 pandemic. We found the score of psychological symptoms among HSPs who were directly exposed (treatment and health cadre) to COVID-19 patients is more than indirectly exposed (support) HSPs. The COVID-19 pandemic has disrupted the mental health status of HSPs and the need for mental health services is increasing.

## Recommendations

This study highlights the demand for high-quality services and evidence-based interventions for the HSPs’ well-being during the COVID-19 pandemic. This study also showed the need for support systems and coping strategies that may assist to decrease stress, PTSD, and depression among HSPs [55].

## Data Availability

The data that support the findings of this study are available from the corresponding author upon reasonable request.

## Abbreviations

HSP: Health service provider
COVID-19: Coronavirus disease 2019
GAD: General anxiety disorder
PTSD: Post-traumatic stress disorder
MDD: Major depression disorder
PHQ: Patient health questionnaire
